# Very-Low-Density Lipoprotein of Metabolic Syndrome Modulates Gap Junctions and Slows Cardiac Conduction

**DOI:** 10.1038/s41598-017-11416-5

**Published:** 2017-09-21

**Authors:** Hsiang-Chun Lee, Chih-Chieh Chen, Wei-Chung Tsai, Hsin-Ting Lin, Yi-Lin Shiao, Sheng-Hsiung Sheu, Bin-Nan Wu, Chu-Huang Chen, Wen-Ter Lai

**Affiliations:** 1 Division of Cardiology, Department of Internal Medicine, Kaohsiung Medical University Hospital, Kaohsiung, Taiwan; 2 Department of Internal Medicine, Faculty of Medicine, College of Medicine, Kaohsiung Medical University, Kaohsiung, Taiwan; 3 Center for Lipid Biosciences, Kaohsiung Medical University Hospital, Kaohsiung, Taiwan; 4 Lipid Science and Aging Research Center, Kaohsiung Medical University, Kaohsiung, Taiwan; 5 Institute/Center of Medical Science and Technology, National Sun Yat-sen University, Kaohsiung, 804 Taiwan; 6 Department of Pharmacology, College of Medicine, Kaohsiung Medical University, Kaohsiung, 807 Taiwan; 7 Graduate Institute of Medicine, College of Medicine, Kaohsiung Medical University, Kaohsiung, Taiwan; 8 Vascular and Medicinal Research, Texas Heart Institute, Houston, TX USA

## Abstract

Very-low-density lipoproteins (VLDL) is a hallmark of metabolic syndrome (MetS) and each manifestation of MetS is related to atrial fibrillation (AF) risks. Slowed atrial conduction is a mechanism of AF in MetS. We hypothesized that VLDL can modulate and reduce atrial gap junctions. VLDLs were separated from normal (Normal-VLDL) and MetS (MetS-VLDL) individuals. VLDLs (15 µg/g) and equivalent volumes of saline (CTL) were injected respectively to C57BL/6 mice for 6 weeks. Electrocardiograms demonstrated that MetS-VLDL induced prolongation of P wave (P = 0.041), PR intervals (P = 0.014), QRS duration and QTc interval (both P = 0.003), but Normal-VLDL did not. Optical mapping of perfused hearts confirmed slowed conduction on atria and ventricles of MetS-VLDL mice. Slowed cardiac conduction was associated with significant atrial and ventricular remodeling, along with systolic dysfunction and comparable intra-cardiac fibrosis. MetS-VLDL induced downregulation of Cx40 and Cx43 at transcriptional, translational and tissue levels, and it also enhanced O-GlcNAcylation of Cx40 and Cx43. Protein structure analyses predicted O-GlcNAcylation at serine 18 of Cx40 and Cx43 which may impair stability of gap junctions. In conclusion, MetS-VLDL modulates gap junctions and delays both atrial and ventricular conduction. VLDL may contribute to the pathophysiology of atrial fibrillation and ventricular arrhythmias in MetS.

## Introduction

Metabolic syndrome (MetS) is not only an important risk factor of atrial fibrillation (AF) but also increases the morbidity and mortality for patients with AF. For aging population, the incidence of AF is increasing, along with its complications such as thromboembolism, stroke, and heart failure causing disabilities, morbidity and mortality^1^. Despite decades of extensive researches, prevention of AF remains a challenge.

One of the key components of MetS is dyslipidemia which manifests with reduced high-density lipoprotein and elevated triglycerides, which is mostly carried by very-low density lipoprotein (VLDL) in blood circulation. In our previous study, VLDL of individuals with MetS (MetS-VLDL) was found different from VLDL of healthy individuals (Normal-VLDL), in terms of abundance of electronegativity and apolipoprotein C-rich fraction. MetS-VLDL exerts substantially larger internalization and cytotoxicity to endothelial cells and atrial myocytes^2,3^. *In vivo*, MetS-VLDL induces atrial remodeling and vulnerability of AF in our VLDL-injection mouse model^3^. The mechanisms warrant further studies.

Electrical conduction occurring along atria from the sinus node to the ventricles can be reflected by the PR interval in electrocardiography. The Framingham heart study has defined prolonged PR interval as a risk factor for AF^4^. PR prolongation is also associated with adverse outcomes such as incidence of pacemaker implantation, and all-cause mortality^5,6^. Clinical studies suggest that PR interval prolongation is related to obesity, increased waist circumference, and components of MetS^7^. Electrophysiological causes of PR interval prolongation include intra-atrial conduction defects, conduction disease in the AV node, conduction disease in the His Bundle, increased vagal tone and the presence of medicine leading to increased AV nodal conduction delay^8^. In addition to PR interval, prolongation of P wave duration was also found robustly associated with an increased risk of AF in a large primary care population^9^. The mechanisms underlying atrial conduction defect in MetS remain unclear.

Accumulating evidence in animal studies suggests that there is a link between gap junction changes and AF^10–14^. The gap junction proteins Cx40 and Cx43 that are encoded by gene GJA5 and GJA1 respectively, these together form the low resistance pathways for electrical activity between atrial cardiomyocytes and are responsible for coordinated electrical activity in the atria. Changes in Cx40 expression and distribution in the atrial myocardium have been shown in parallel with reduced atrial effective refractory period, enhanced spatial dispersion of atrial refractoriness, or abnormal atrial impulse conduction^15^. In addition, GJA5 mutations or polymorphisms bring an inherited predisposition to AF^16^.

Post-translational modifications are also important for connexins regulation^17^. The O-GlcNAcylation of connexins is a posttranslational, and reversible modification that involves the addition of N-acetylglucosamine (GlcNAc) on serine or threonine residues. O-GlcNAcylation can also regulate the phosphorylation of Cx40 and Cx43. Evidence has shown a contribution of O-GlcNAcylation to endothelial dysfunction in diabetes mellitus and in hyperglycemia-associated cardiovascular defects^18,19^. The O-GlcNAcylation of Cx40 or Cx43 in atrial myocytes has not been explored.

The modulation of gap junctions on the occurrence of AF has not been examined in the MetS model. In our VLDL-injection mouse model, the atrial remodeling and AF susceptibility to sympathetic stimulation are observed with injection of MetS-VLDL, but not with injection of Normal-VLDL^3^. Using this model, the study was aimed to examine the hypothesis that MetS-VLDL can modulate atrial connexins expression and slow atrial conduction. Whether MetS-VLDL induces O-GlcNAcylation of Cx40 and/or Cx43 in atrial myocytes and whether O-GlcNAcylation would change the gap junction stability were also assessed. The results are of general importance with respect to pathogenesis of AF in MetS.

## Results

### *In vivo* MetS-VLDL resulted in prolonged P waves, PR intervals, QRS width, and QT intervals

**Figure 1:**
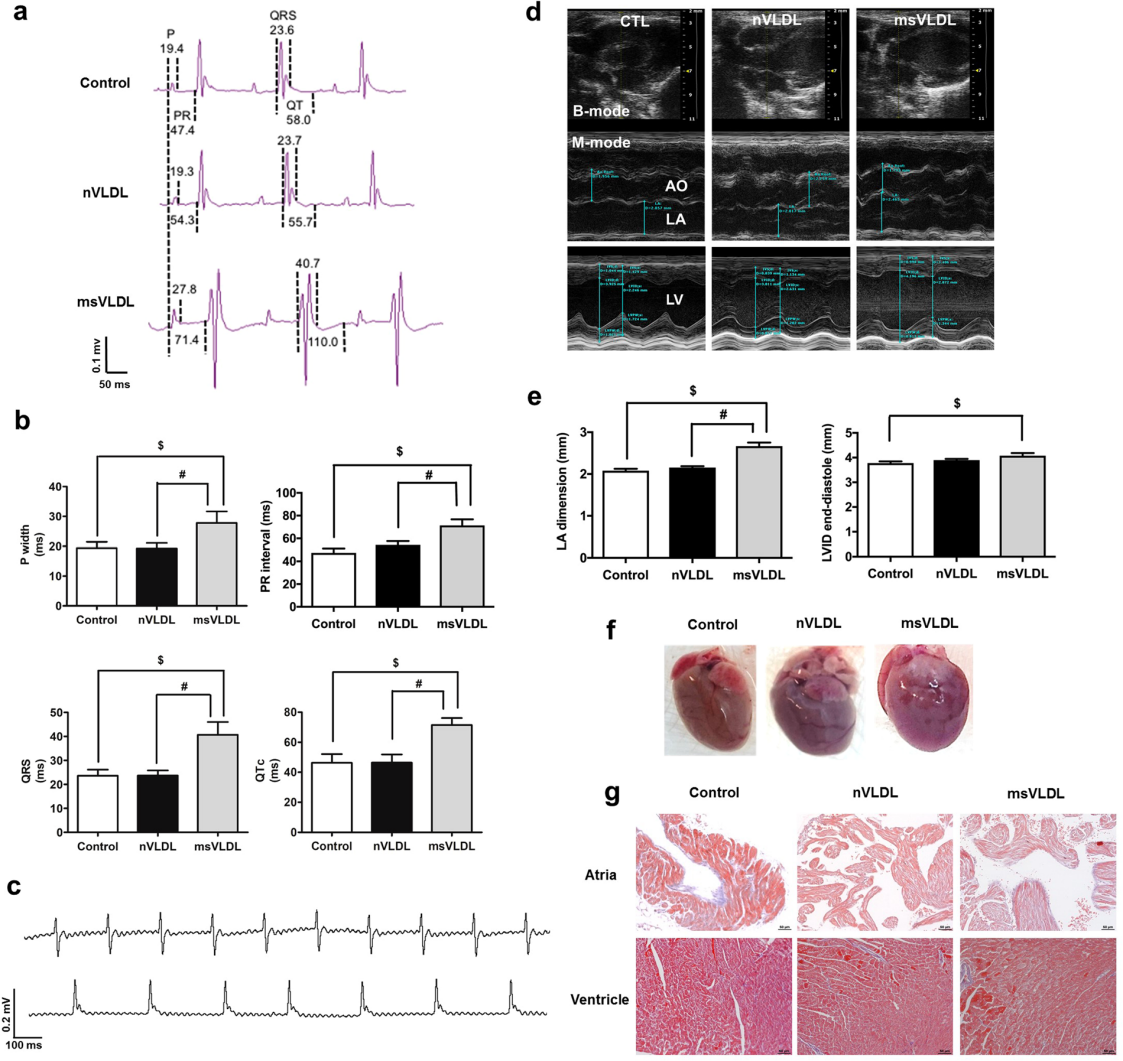
Prolongation of P waves, PR intervals, QRS, and QT intervals along with Left atrial and left ventricular dilatation and comparable intra-cardiac fibrosis. (**a**) The dash lines indicate measurements of P wave width (P), PR interval (PR), QRS width, and QT interval. (**b**) P, PR, QRS, and QTc in MetS-VLDL injected mice (msVLDL), Normal-VLDL injected mice (nVLDL) and control mice (n = 10 for each) ^$^P < 0.05; ^$^P < 0.05. (**c**) Representative electrocardiographic tracings of unprovoked atrial fibrillation from two msVLDL mice. (**d**) B-mode and M-mode images for measurements of dimensions of aortic root (AO), left atria (LA), and left ventricles (LV). (**e**) LA size and LV end-diastolic dimension (msVLDL vs control, ^$^P < 0.01; msVLDL vs nVLDL, ^#^P < 0.01). (**f**) Gross morphology of hearts. The msVLDL atrium was larger than the control and nVLDL ones. (**g**) Histological sections of control, nVLDL, and msVLDL stained with Masson-Trichrome; black bars indicate 50 μm.

To determine the *in vivo* effect of VLDL on intra-cardiac electrical conduction, we used surface ECG to determine parameters of P wave width, PR intervals, QRS width, and QT intervals of three groups mice with either saline (control), Normal-VLDL (nVLDL), and MetS-VLDL injection (msVLDL) (Fig. 1a and b, Table 1). Similar to human ECG, the murine ECG shows P waves, reflecting spread of atrial depolarization from impulse of sinoatrial node. Subsequently, the PR interval reflects impulse conduction from the atria through the AV node and the His-bundle to the ventricles. Then the QRS complex reflects ventricular depolarization. The QT interval was defined as the period between the initiation of the Q wave to the point of T waves returning to the isoelectrical baseline. The murine QTc interval is corrected with heart rate using Bazett’s formula, which is commonly used in human ECG analysis^20^. In msVLDL mice (n = 10), P wave was significantly wider than nVLDL (n = 10) and control mice (n = 10) (Control 19.4 ± 6.4 vs nVLDL 19.3 ± 5.6 vs msVLDL 27.8 ± 12.3 msec, ANOVA P = 0.041; ^$^p = 0.01, ^#^p = 0.03; Fig. 1b). The PR interval was significantly prolonged in msVLDL mice (Control 47.4 ± 12.2 vs nVLDL 54.3 ± 10.6 vs msVLDL 71.4 ± 16.8 msec, ANOVA P = 0.015; ^$^P < 0.01, ^#^P = 0.03; Fig. 1b). The msVLDL also had wider QRS (^$^P < 0.05, ^#^P < 0.01; Fig. 1b) and QT prolongation (^$^P < 0.01, ^#^P < 0.01; Fig. 1b). In summary, these findings indicate that MetS-VLDL delayed intra-atrial, atria-ventricular, and ventricular conduction and ventricular repolarization. Moreover, unprovoked AF was only observed in msVLDL mice after 6 week’s MetS-VLDL injection and before sacrifice (Fig. 1c).

**Table 1:** Electrocardiographic parameters after six weeks of VLDLs injection.

	Control (n = 10)	nVLDL (n = 10)	msVLDL (n = 10)	p value
BW (g)	27.3 ± 1.4	29.1 ± 1.9	27.3 ± 2.3	0.0678*
HR (bpm)	355.5 ± 98.2	406.4 ± 60.3	345.5 ± 112.6	0.3068
P width (msec)	19.4 ± 6.4	19.3 ± 5.6	27.8 ± 12.3	0.0414^¶#^
P amplitude (mV)	0.053 ± 0.024	0.051 ± 0.027	0.082 ± 0.016	0.0068^¶#^
PR (msec)	47.4 ± 12.2	54.3 ± 10.6	71.4 ± 16.8	0.0140^¶#^
QRS (msec)	23.6 ± 8.1	23.7 ± 6.7	40.7 ± 16.9	0.0028^¶#^
QT (msec)	58.0 ± 27.3	55.7 ± 22.2	110.0 ± 41.7	0.0008^¶#^
QTc (msec)	46.3 ± 18.2	46.4 ± 17.1	71.5 ± 14.7	0.0025^¶#^

BW, body weight; HR, heart rate; P, P wave; PR, PR intervals; QRS, QRS durations; QT, QT intervals; QTc, corrected QT intervals. Data are mean ± SD. *Comparison significant for nVLDL versus Control. ^¶^Comparison significant for msVLDL versus Control. ^#^Comparison significant for msVLDL versus nVLDL.

### MetS-VLDL induced left atrial and ventricular dilation and impaired left ventricular systolic function without enhancing cardiac fibrosis

The cardiac structure and left ventricular function were examined by echocardiography after 6 week’s VLDL injection (Fig. 1d and e, and Table 2). Although both VLDLs seemed to increase LV mass (ANOVA P = 0.4372), only MetS-VLDL induced significant dilatation of left atria (P < 0.0001 vs control and P < 0.0001 vs nVLDL) and left ventricles (P = 0.0189 vs control). The dilated left ventricular diameter and volume in msVLDL mice hearts were associated with reduced left ventricular ejection fraction (EF, msVLDL 60.3 ± 7.3% vs control 67.6 ± 7.9%; P = 0.0086) and fraction shortening (FS, msVLDL 32.0 ± 4.9% vs 37.4 ± 6.2%; P = 0.0096). These results indicate that MetS-VLDL induced significant atrial and ventricular remodeling along with impaired left ventricular systolic functions.

**Table 2:** Echocardiographic parameters in aged mice after six weeks of VLDL injection.

	Control (n = 18)	nVLDL (n = 17)	msVLDL (n = 19)	*P* value
HR (bpm)	425 ± 28	424 ± 46	410 ± 67	0.7243
**Measurement** (mm)				
Ao Root	1.91 ± 0.12	1.99 ± 0.10	1.98 ± 0.14	0.0992
LA	2.15 ± 0.17	2.23 ± 0.25	2.62 ± 0.33	<0.0001^¶#^
IVSd	1.03 ± 0.16	1.04 ± 0.12	1.03 ± 0.14	0.9307
LVIDd	3.78 ± 0.27	3.90 ± 0.20	4.08 ± 0.46	0.0338^¶^
LVPWd	0.93 ± 0.13	0.93 ± 0.16	0.86 ± 0.12	0.2253
LVIDs	2.37 ± 0.33	2.50 ± 0.24	2.79 ± 0.46	0.0038^¶^
**Calculation**				
EF (%)	67.6 ± 7.9	66.0 ± 5.8	60.3 ± 7.3	0.0076^¶^
FS (%)	37.4 ± 6.2	36.0 ± 4.4	32.0 ± 4.9	0.0093^¶^
LV Mass (mg)	142.2 ± 23.6	151.1 ± 23.2	152.6 ± 29.4	0.4372
LVEDV (µL)	61.6 ± 9.7	66.1 ± 8.0	74.5 ± 19.6	0.0222^¶^
LVESV (µL)	20.2 ± 6.5	22.6 ± 5.1	30.4 ± 11.9	0.0019^¶#^

HR, heart rate during the echocardiographic measurement; Ao Root, aortic root diameter; LA, left atrium diameter; IVSd, end-diastolic interventricular septum thickness; LVIDd, end-diastolic LV internal dimension; LVPWd, end-diastolic LV posterior wall thickness; LVIDs, end-systolic LV internal dimension; EF, ejection of fraction; FS, fraction of shortening; LV, left ventricle; LVEDV, LV end-diastolic volume; LVESD, LV end-systolic volume. ^¶^Comparisons significant for msVLDL vs Control. ^#^Comparison significant for msVLDL vs nVLDL.

After hearts were dissected and washed, the atria and ventricles were grossly larger for the msVLDL groups (Fig. 1f). Increased fibrosis in myocardium can slow myocardial conduction. Therefore, Mason-Trichrome staining of the atrial and ventricular tissues was performed and the fibrosis region was quantified by ImageJ software. Degree of fibrosis was comparable among groups (Fig. 1g). Therefore, the delayed atrial and ventricular conduction in msVLDL mice is not related to intra-cardiac fibrosis.

### *In vivo* downregulation of Cx40 and Cx43 mRNA and protein induced by MetS-VLDL

**Figure 2:**
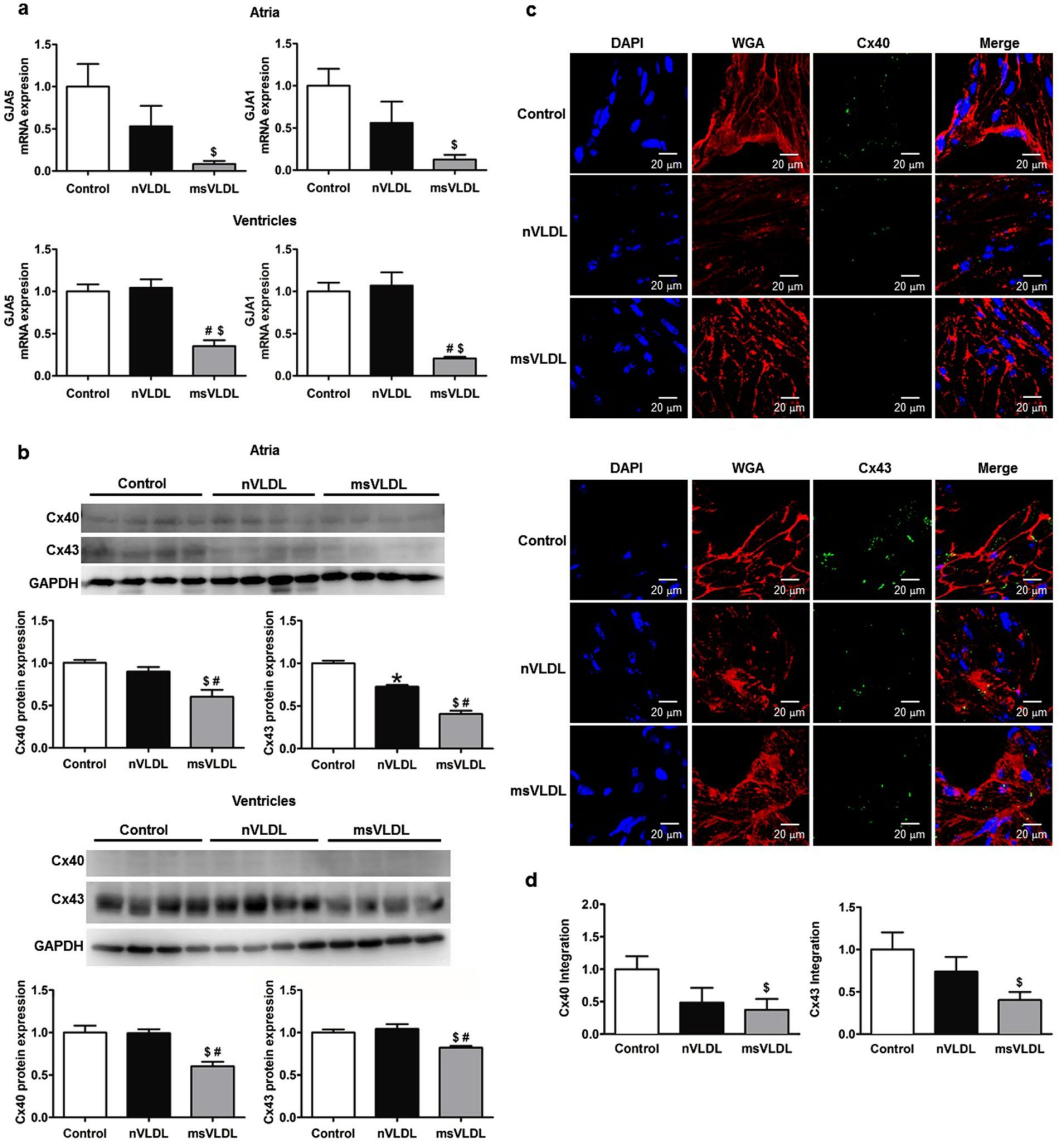
The transcriptional, translational and tissue expression of Cx40 and Cx43. (**a**) Results of real-time PCR of GJA5 and GJA1 in atrial and ventricular tissues. For atria (n = 3 for each), GJA5 ^$^P = 0.0028 and GJA1 ^$^P = 0.014 for GJA1; msVLDL vs control. For ventricles (n = 5 for each), GJA5 ^#^P = 0.0005, msVLDL vs nVLDL; ^$^P = 0.0003, msVLDL vs control; GJA1 ^#^P = 0.0006 and ^$^P < 0.0001). (**b**) Western blotting for atrial and ventricular expression of Cx40 and Cx43. For atria Cx40 (n = 4 for each), msVLDL vs control, ^$^P = 0.004; msVLDL vs nVLDL, ^#^P = 0.023. For atria Cx43 (n = 4 for each), msVLDL vs control, ^$^P < 0.0001; msVLDL vs nVLDL, ^#^P = 0.0004; nVLDL vs control, *P = 0.0003. In msVLDL ventricles, Cx40 and Cx43 protein reduction is significant (^#^P = 0.0015 and ^#^P = 0.01 vs nVLDL; ^$^P = 0.0067 and ^$^P = 0.0045 vs control). (**c**) Immunohistochemistry of atrial tissues (n = 7 for each). Nuclei with DAPI staining appear blue. The plasma membrane with WGA staining appear red. Cx40 (upper panel) and Cx43 (lower panel) appear green. The scale bars indicate 20 µm. (**d**) The quantification of Cx40 and Cx43 signaling integrated from a series of confocal image sections (Cx40 and Cx43 integration). For msVLDL, Cx40 and Cx43 (0.376 ± 0.164 and 0.403 ± 0.285 folds to control) are significantly reduced (^$^P = 0.044 and ^$^P = 0.043 vs control).

Decreased gap junction function can potentially slow myocardial conduction. Therefore, we examine if expression of cardiac connexins can be reduced by MetS-VLDL. Atrial and ventricular expression of GJA5, and GJA1 mRNA were determined by real-time PCR (Fig. 2a). Protein levels of Cx40 and Cx43 were determined by Western blotting (Fig. 2b). For atria, GJA5 and GJA1 mRNA was decreased in msVLDL (0.083 ± 0.062 and 0.127 ± 0.096 folds to control, n = 3), nonsignificant when compared to nVLDL (0.530 ± 0.421 and 0.561 ± 0.436 folds to control, n = 3; P = 0.143 and P = 0.168) and significantly compared to control mice (msVLDL vs conrtol, P = 0.0028 for GJA5, P = 0.014 for GJA1). For ventricles, GJA5 and GJA1 mRNA was significantly decreased in msVLDL (0.352 ± 0.155 and 0.207 ± 0.044 folds to control, n = 5), compared to nVLDL (1.043 ± 0.225 and 1.069 ± 0.351 folds to control, n = 5; P = 0.0005 and P = 0.0006), and control (P = 0.0003 and P < 0.0001). The protein levels of Cx40 and Cx43 were significantly decreased in msVLDL atria (0.60 ± 0.17 and 0.40 ± 0.08 folds to control, n = 4), compared to nVLDL group (0.90 ± 0.11 and 0.72 ± 0.04 folds to control, n = 4; P = 0.023 and P = 0.0004), and the control (P = 0.004 and P < 0.0001). In ventricles, the protein levels of Cx40 and Cx43 were also significantly decreased in msVLDL mice (0.60 ± 0.11 and 0.82 ± 0.04 folds to control, n = 4), compared to nVLDL group (0.99 ± 0.09 and 1.05 ± 0.11 folds to control, n = 4; P = 0.0015 and P = 0.01), and the control (P = 0.0067 and P = 0.0045). *In vivo* effect of VLDLs on atrial expression of Cx40 and Cx43 was further assessed by the immunohistochemistry study (Fig. 2c and d). In msVLDL atria, Cx40 and Cx43 were significantly down-regulated (0.376 ± 0.164 and 0.403 ± 0.285 folds to control, P = 0.043 and P = 0.044 vs control); whereas Cx40 and Cx43 changes in nVLDL were nonsignificant (0.486 ± 0.227 and 0.740 ± 0.104 folds to control, P = 0.165 and P = 0.164 vs control). These findings indicate that MetS-VLDL substantially reduced atrial Cx40 and Cx43 expression at transcriptional, translational, and tissue levels, suggesting the pathogenic role of Cx40 and Cx43 reduction on delayed atrial conduction and development of AF in msVLDL mice.

### MetS-VLDL induced delayed atrial and ventricular conduction

**Figure 3:**
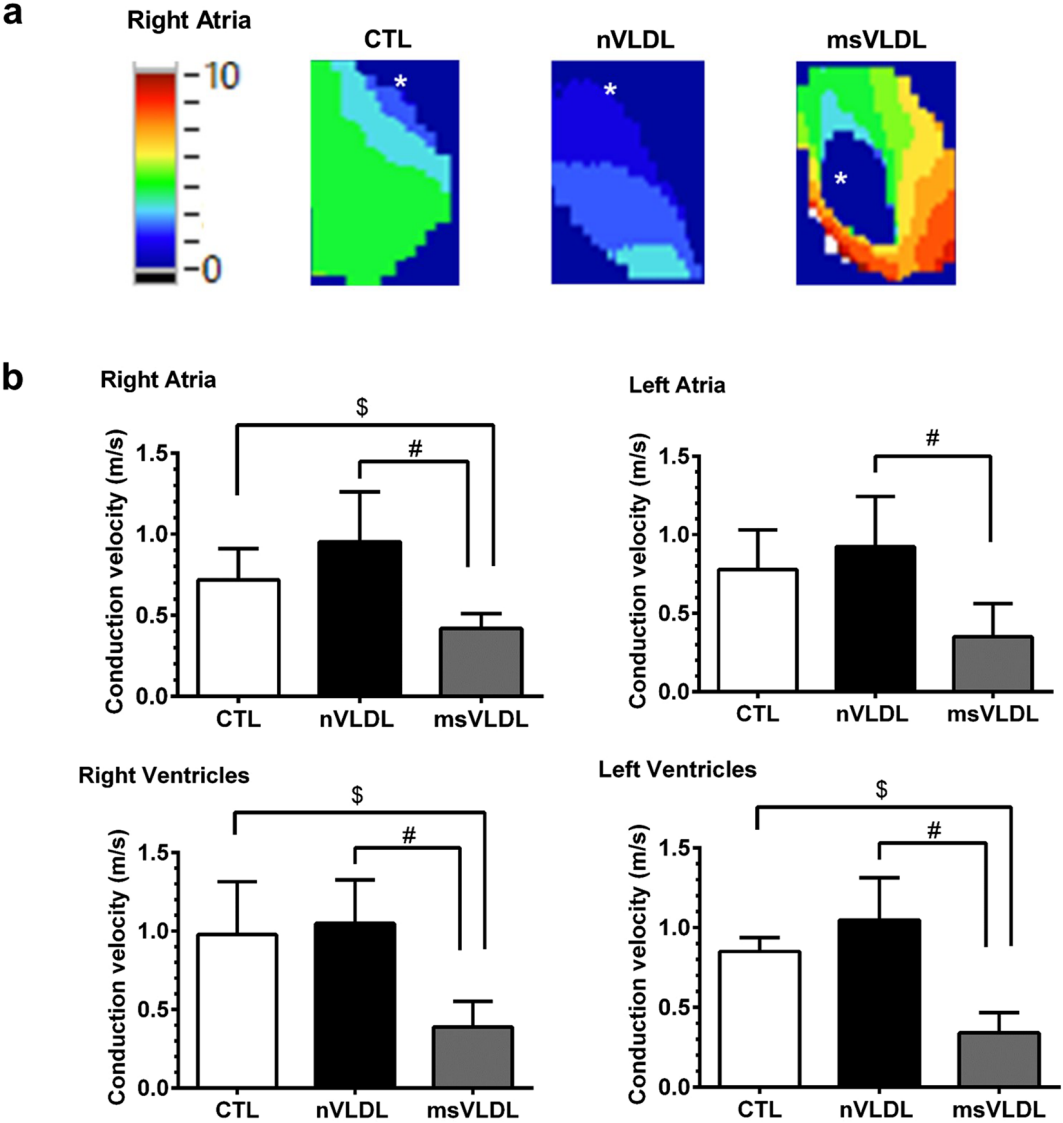
Atrial and ventricular conduction evaluated by optical mapping in CTL, nVLDL, and msVLDL mice. (**a**) The right atrial propagations in CTL, nVLDL, and msVLDL during rapid atrial pacing (pacing cycle length = 200 ms) were demonstrated in the isochronal map. The white asterisk indicates the pacing site. (**b**) The conduction velocity was compared among CTL, nVLDL, and msVLDL groups. The conduction velocity in RA, RV and LV were all slower in msVLDL than in CTL and nVLDL mice. Although the conduction velocity in LA is slower in msVLDL than in nVLDL mice, there is no difference in conduction velocity comparing LA in msVLDL than CLT group. ^$^P < 0.05 compared with CTL. ^#^P < 0.05 compared with nVLDL.

To evaluate if reduced Cx40 and Cx43 co-exist with slower intra-cardiac conduction velocity, the optical mapping technique was used to image the membrane potential of intact atria and ventricles on *ex-vivo* perfused hearts^21^. The membrane potential propagations and the derived conduction velocity of CTL, nVLDL, and msVLDL *ex-vivo* hearts during rapid atrial pacing (pacing cycle length = 200 ms) were demonstrated in the Fig. 3. The conduction velocity in atria and ventricles were all slower in msVLDL compared with CTL and nVLDL mice (right atria P = 0.0123; left atria, P = 0.0222; right ventricles P = 0.0091; left ventricle, P = 0.0004 by ANOVA analysis).

### O-GlcNAcylation of Cx40 and Cx43 in atrial myocytes was induced by MetS-VLDL but not by Normal-VLDL

**Figure 4:**
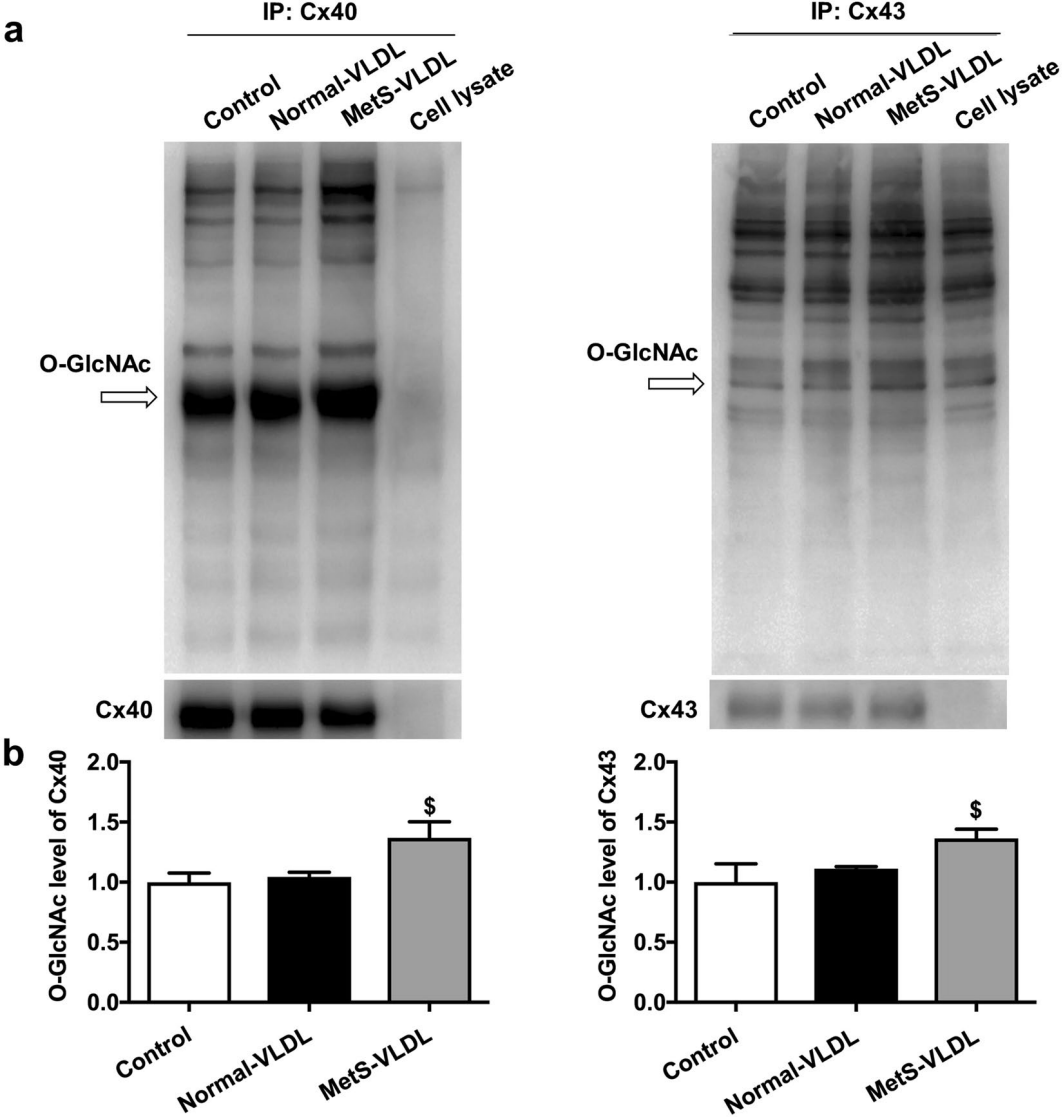
The MetS-VLDL induced O-GlcNAcylation of Cx40 and Cx43. (**a**) The immunoblot shows increase in total O-GlcNAcylated Cx40 and Cx40 proteins in MetS-VLDL group. (**b**) Densitometry demonstrates that in the MetS-VLDL group, a significant increase in O-GlcNAcylation levels for both Cx40 and Cx43 (n = 4 for each group; 1.37 ± 0.26 folds to control, ^$^P = 0.03 for Cx40 and 1.36 ± 0.15 folds to control, ^$^P = 0.02 for Cx43).

It is known from Cx40^+/−^ mice that the PR interval and atrial conduction velocity is not delayed with a 50% reduction of Cx40 protein expression^22,23^. Except for the expression level, posttranslational modification plays a critical role in determining gap junction function. We hypothesized that MetS-VLDL can induce O-GlcNAcylation of Cx40 or Cx43 in cardiomyocytes. Atrial myocytes HL-1 were cultured and treated with either MetS-VLDL or Normal-VLDL of 25 mg/L for 16 hours. The immunoprecipitation revealed that Cx40 and Cx43 were markedly O-GlcNAcylated in the MetS-VLDL group (Cx40, P = 0.0313 vs control; Cx43, P = 0.017 vs control) (Fig. 4); whereas Normal-VLDL did not increase O-GlcNAcylation of Cx40 and Cx43 at the same concentration with the same duration of treatment.

### Molecular modeling and structural analysis of human Cx40 and Cx43

**Figure 5:**
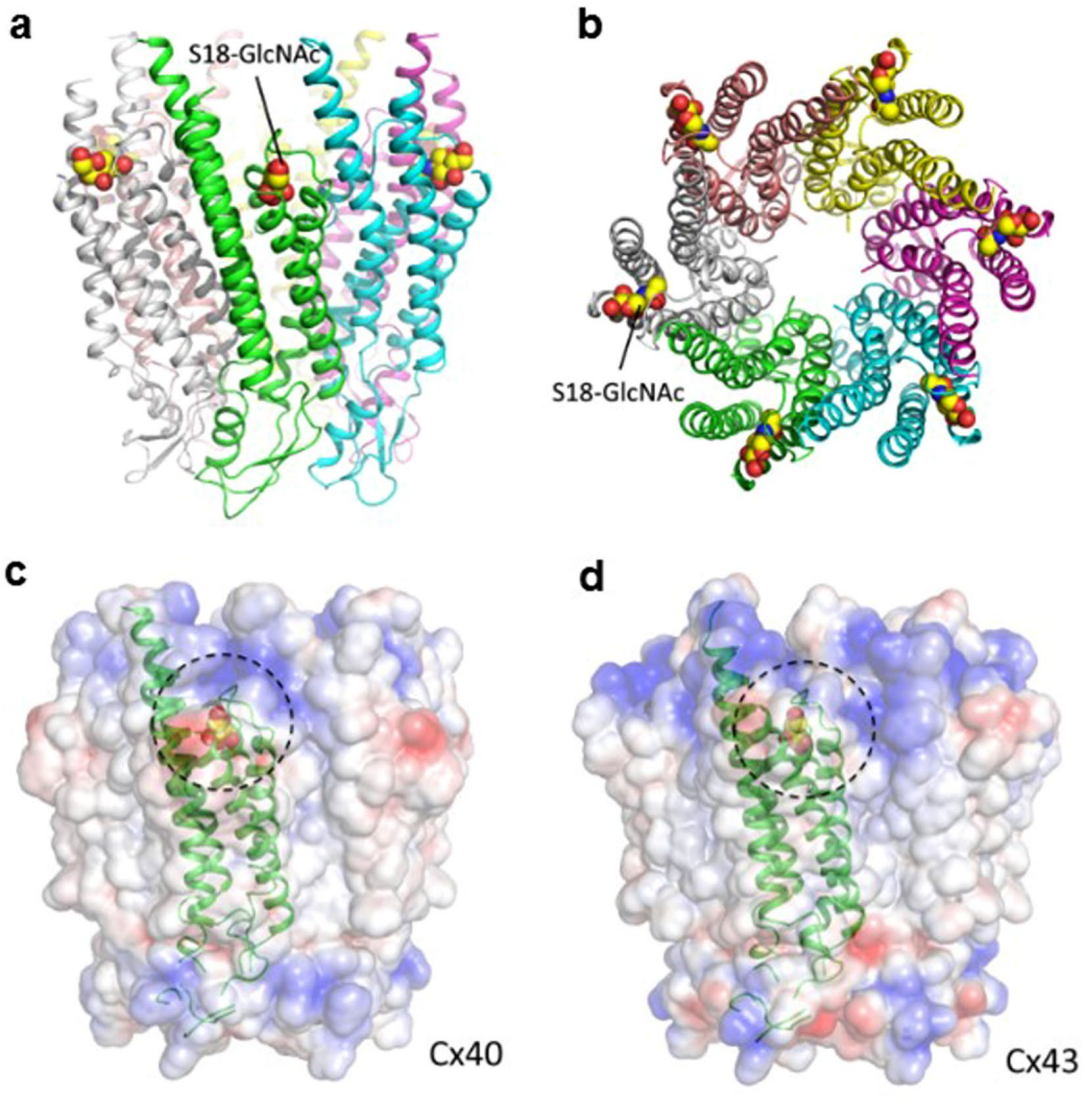
Structure of the O-GlcNAcylated Cx40 and Cx43 proteins and their electrostatic potential surface. (**a**) The side view of Cx40 structure with O-GlcNAcylation at the residue Serine 18. (**b**) The top view of Cx40 with O-GlcNAcylation. (**c**,**d**) The electrostatic potential surface of Cx40 and Cx43 with O-GlcNAcylation. The O-GlcNAc on the surface is highlighted with dotted circles. Electrostatic potential surfaces are blue for positive, white for hydrophobic, and red for negative. All images were generated by the PyMOL (Available: http://www.pymol.org).

Next, the computational modeling was used to test the hypothesis that O-GlcNAcylation modification of Cx40 and Cx43 can affect the gap junction stability. Firstly, human Cx40 and Cx43 oligomerization models were constructed using the published crystal structure Cx26 gap junction (PDB code 2ZW3) as the template (Fig. 5). As the result, connexins oligomerize into hexamers and the connexin was predicted to have four trans-membrane helices and two extracellular loops, which are thought to contain a β-strand structure and are an essential structural basis for the docking of two connexons to form the complete gap junction channel^24^. Then the O-GlcNAcylation site prediction was performed. Both sequence-based (OGTSite) and structure-based (Glycoprotein Builder) analyses predicted the most possible O-GlcNAcylation sites of Cx40 and Cx43 at the residue Serine 18, which is also located at the interface between the connexins. This finding suggested that the O-GlcNAcylation of Serine 18 may disturb stability between connexins interface and thus hamper the oligomerization of connexins into connexons.

## Discussion

The key findings of this study are as follows: (1) MetS-VLDL can significantly reduce Cx40 and Cx43 mRNA and protein expression in atrial and ventricular tissues. (2) MetS-VLDL can induce modification of O-GlcNAcylation on Cx40 and Cx43 connexins. (3) MetS-VLDL slowed atrial and ventricular conduction, and delay ventricular repolarization; whereas Normal-VLDL did not exert the aforementioned findings. (4) The O-GlcNAcylation of Cx40 was predicted at the residue Serine 18, and as a result the stability between adjacent connexins binding may be disturbed. Noteworthy, the delayed cardiac conduction in msVLDL mice is concomitant with significant atrial and left ventricular remodeling and systolic dysfunction, whereas are not related to intra-cardiac fibrosis. This study is extended from our previous works that identified remarkable differences between MetS-VLDL and Normal-VLDL on inducing AF vulnerability^2,3^, and the key findings may further explain early pathogenesis of AF in MetS.

Cardiac electrophysiology in mouse models has been well validated. The normal range for mice PR interval is 30–56 msec and for QRS duration is 9–30 msec^20^. In this study, values for the control were consistent and within the normal ranges. Mice with MetS-VLDL injection had values longer than the normal limits, but those with Normal-VLDL did not. Similarly, QT and QTc intervals were prolonged in msVLDL mice and were normal in control and nVLDL mice which were consistent to previous studies^20,25,26^. Also, consistent with a recent delicate work using high-fat-diet mice by Takahashi *et al*., prolonged P wave duration was also observed and it was shown associated with downregulation of Cx40 and atrial tachycardia inducibility^27^. However, PR interval and QRS duration in their high-fat-diet mouse model were not prolonged.

Cx43 is expressed in cardiomyocytes throughout the heart, whereas Cx40 expression is exclusively to the atria and the His bundle and Purkinje fibers^28^. Deficiencies in either Cx43 or Cx40 result in cardiac conduction defects^28^. For atrial tachyarrhythmia, Cx40 downregulation was suggested more important than Cx43^11,29^. Studies have shown that P wave duration is not affected with reduced Cx43 levels in Cx43^+/−^ mice^30^. Re-distribution of Cx43, in which Cx43 is moved from the primary location of intercalated disc between adjacent cardiomyocytes to disperse throughout the sarcolemma, has been suggested more closely related to cardiac conduction delay^11,31^. For Cx40, conflicting results have been reported with respect to the effect of Cx40 deficiency on atrial conduction^29^. With a 50% reduction of Cx40 protein in Cx40^+/−^ mice, PR interval and atrial conduction velocity was not significantly changed^22,23^. The aforementioned works help to explain why nVLDL mice, in which with an approximate 30% reduction of atrial Cx40 expression, had unaffected durations of P wave and PR intervals.

Oligomerization of connexins into hemichannel (the hexamer, connexon) takes place after the protein exits the endoplasmic reticulum. Once delivered to the cell surface, a pair of connexons from neighboring cells form gap junction plaques. The half-life of gap junctions is short (<5 h) and both proteasomal and lysosomal degradation of gap junctions are thought involved in connexin degradation^32^. O-GlcNAcylation of connexins may affect the assembly of connexons and resulting change of gap junction turnover. In endothelial cells under high-glucose condition, and O-GlcNAcylated Cx40 was shown to cause gap junction dysfunction and resulted in endothelial dysfunction^19^. It is possible that O-GlcNAcylation directly affects gating of gap junction channels or indirectly through competing for a phosphorylation of adjacent residues, and/or impairs stability of gap junction structure. To our knowledge, this study for the first time suggested an important role of O-GlcNAcylation for cardiac gap junctional function. We predicted the O-GlcNAcylated residues for Cx40 and Cx43. This prediction is crucial to determine further detail mechanism such as the biophysical impact of O-GlcNAcylated residue to the gap channel gating. Certainly, additional work is needed to fully establish the connection between Cx40 and Cx43 O-GlcNAcylation and MetS-VLDL-induced delayed atrial and ventricular conduction in this mouse model. The application of either the gap junctional coupling estimation with a dye transfer or the trans-junctional voltage-gating property of cells pairs measured on the dual whole-cell voltage-clamp technique, could shed some light on the impact of O-GlcNAcylation on Cx40 and Cx43 gap junctional function.

Although the regulation of circulatory VLDL concentration is complex, elevated VLDL is a hallmark of the MetS. A large-scale clinical study reported that circulatory lipoprotein subclasses including VLDL particles are modified in individuals with abnormal glucose tolerance and newly diagnosed diabetes mellitus^33^. On the other hand, another clinical study with mean follow-up of 9.6 years identified high-density lipoprotein cholesterol and triglycerides as independent risk factors for AF incidence^34^. Several clinical studies and meta-analysis consistently indicated a reduced risk of AF in patients treated with statins and the mechanisms of the beneficial effects remain uncertain^35^. These clinical evidences all indicate that circulatory VLDL may be crucial in AF pathogenesis. However, we could not find any report evaluating the correlation electrocardiography P wave or LA remodeling with circulatory VLDL in human beings. The unnoticed role of VLDL in MetS-related cardiovascular disease may be due to an overlook of differences between MetS-VLDL and Normal-VLDL. Further clinical and translational studies are required to elucidate the putative mechanism in which VLDL induces atrial and ventricular remodeling in MetS.

Upon our main research hypothesis for this study, the delayed ventricular conduction and repolarization in msVLDL mice were additional findings. Consistent with our previous study, significant cardiac remodeling became phenomenal after 6 week’s MetS-VLDL injection. Compared with our previous published data done with young mice (14-week-old), the aged mice (11-month-old) had larger atrial and ventricular sizes and larger ventricular mass^3^. In aged mice, MetS-VLDL significantly induced not only left atrial dilation, but also left ventricular dilation associated with reduced left ventricular ejection fraction. These observations suggest that MetS-VLDL exerts more cardiac lipotoxicity to aged mice than to young mice. From clinical observational studies, the association of QRS and QT prolongation with MetS has been noticed^36,37^. It is well known that prolonged QRS duration and QT prolongation are risk factors for sudden cardiac death. The adverse left ventricular remodeling, systolic dysfunction, and coexisting delayed ventricular conduction and repolarization in the msVLDL mice suggest an important role of VLDL in pathophysiology of ventricular arrhythmias in individuals with MetS. Whether our msVLDL mice are vulnerable to ventricular arrhythmias remains undetermined.

This study used human VLDL isolated from MetS patients and normal individuals to inject into immune-competent mice for 6 weeks. Although the VLDL and the VLDL receptor are similar and quite conserved in the human beings and mice, it is certainly that immune response was developed in these mice against foreign (human) along with the injection. The possibility that mice develop different immune responses on MetS-VLDL and Normal-VLDL cannot be excluded. In addition, some parameters of putative interest were unavailable, such as determination of VLDL particle size, VLDL levels after injection, and lipidomics of VLDL samples that were used in this study.

In conclusion, this study highlights evidence that MetS-VLDL *in vivo* and *in vitro* modulates cardiac gap junctions Cx40 and Cx43. Delayed atrial and ventricular conduction is induced by MetS-VLDL, at least in part, via its distinct effect on substantial decrease in Cx40 and Cx43 expression, and on enhancing the O-GlcNAcylation modification. We suggest that the MetS-VLDL plays a pivotal role in cardiac remodeling and pathogenesis of AF and ventricular arrhythmia. VLDL may be a novel target for prevention or control of AF, particularly as concerning individuals with MetS.

## Methods

### Human VLDL isolation

We followed the Helsinki Declaration principles. The research was approved by the Kaohsiung Medical University Hospital Ethics Review Board. Individuals who either met the criteria of the MetS or otherwise were healthy gave informed consent for blood donations. Then Normal-VLDL and MetS-VLDL (d = 0.930–1.006 g/mL) were isolated by sequential ultracentrifugation as previously described^2,3,38^.

### Mice and diet

To test *in vivo* effects of VLDLs, we injected MetS-VLDL or Normal-VLDL intravenously to mice tail veins at a dose of 15 μg/g, three times a week for 6 consecutive weeks. The 15 μg/g dose was chosen to match the human normal VLDL concentration^38^, which is between 2 and 30 mg/dL. Nine-month-old C57BL/6 male mice from the National Laboratory Animal Center (Taipei, Taiwan) were maintained in a temperature-controlled facility (21 to 22 °C) with a 12-h light/dark cycle. The mice had free access to water and a standard chow diet. After a two-week acclimatization period, control and VLDL-injection groups were created by random separation of mice from the same lot. The control mice were injected with an equivalent 50 μL volume of phosphate buffered saline. Body weights were recorded weekly. Total number of mice used was 63 and all of them received saline or VLDLs injection, in which 18 were for the *ex vivo* optical mapping. All applicable institutional and governmental regulations concerning ethical use of animals were performed confirming to the NIH guidelines and all animal procedures were approved by the Institutional Animal Care and Use Committee of Kaohsiung Medical University.

### Surface electrocardiography and echocardiography

Mice were anesthetized with 1.5–2% isofluorane. Platinum electrodes were inserted subcutaneously in the limbs and connected to a custom-built electrocardiogram (ECG) amplifier and recorded for about 5 minutes at 2 MHz. Raw tracings without clear P waves or T waves were discarded for intervals analysis. The analysis for intervals was done with LabChart 5 software (AD instruments), and QTc intervals were calculated with formula for mice: QTc = QT/(RR/100)^1/2^ ^25^. *In vivo* heart chamber dimensions were measured for each mouse (control, n = 10; nVLDL, n = 10; msVLDL, n = 10) under anesthesiology with 1.5–2% isofluorane by using a Vevo2100 (VisualSonics, Inc., Toronto, Ontario, Canada) small animal instrument as previously described^3^.

### Histology of mice atrial tissue

Mouse hearts were perfused with 4% paraformaldehyde at physiological pressures, blotted dry on tissue paper and weighed. Next, they were embedded in paraffin and sections were stained with Masson’s trichrome according to the manufacturer’s protocol as previously described. Measurements were performed using ImageJ software.

### Confocal analysis of atrial tissue

Indirect immunofluorescent detection of Cx40, Cx43 was performed on frozen cross-sections of the atriums of mice of all experimental groups. Series of sections were fix in −20 °C cold methanol for 5 mins, blocked by 0.5% BSA in PBS and incubated with primary monoclonal antibody goat anti-40 (1:1000, Santa Cruz Biotechnology Inc. USA), rabbit anti-43 (1:1000, cell signaling technology, MA) and Wheat Germ Agglutinin, Texas Red®-X Conjugate (WGA, 1:500, Thermo Fisher Scientific Inc. USA) for 2 hours at 37 °C. The sections were subsequently washed with phosphate-buffered saline (PBS) and then followed the application of secondary anti-rabbit and anti-goat conjugated with FITC-fluorescein CF488A (1:500, Sigma-Aldrich Inc. USA) for 50 mins at room temperature. After washing, the sections were mounted into UltraCruz® Mounting Medium (with DAPI) and view by confocal microscope Olympus FV1000 (Olympus, Japan). Morphometric analyses were conducted on coronal sections using individual cell areas from atria that were traced and integrated with Olympus FV1000 software. In short, images were collected using the ×40 objective lens and zoom 1.0 computer setting so that each pixel represented 0.23 μm. Each image was consisted of 512 × 512 pixels. The projection view of 15 consecutive optical sections taken at interval of 0.53 μm was recorded for quantification analysis.

### Quantitative real-time reverse transcriptase PCR and Western blot

Total RNA was prepared using TRI Reagent (Sigma-Adrich, St Louis, MO), then reverse transcribed (Invitrogen, Carlsbad, CA). Quantitative real-time RT-PCR was performed using an ABI 7500 real-time system (Applied Biosystems, Foster city, CA) and TaqMan Universal Master Mix II (Applied Biosystems, CA) with TaqMan probes GJA1 (Mm00439105_m1, Thermo Fisher Scientific Inc. USA) and GJA5 (Mm00433619_s1, Thermo Fisher Scientific Inc. USA). Protein samples were separated using a SDS-polyacrylamide gel and transferred to a nitrocellulose membrane.

Blots were incubated with anti-Cx40 (1:500 dilution; Santa Cruz), anti-Cx43 (1:1000 dilution; Cell Signal), and anti-phospho-Cx43 (1:1000 dilution; Cell Signal), then with horseradish peroxidase-linked secondary antibody. The immunoblots were identified with SuperSignal West Picochemiluminescent substrate (Thermo Fisher Scientific). Band intensity was calculated using ImageJ (National Institutes of Health). Intensity data from cytoplasmic and membraneous proteins in interest were normalized to α-tubulin and pan-cadherin, respectively.

### Langendorff heart preparation

The mice were intraperitoneally injected with 200 units of heparin and anesthetized with sodium pentobarbital (35 mg/kg). After a median sternotomy, the hearts were harvested through a thoracotomy in ice-cold Tyrode’s solution (in mmol/L: NaCl 125, KCl 4.5, NaHCO3 24, NaH2PO4 1.8, CaCl2 1.3, MgCl2 0.5, and glucose 5.5) and cannulated via the aorta. The heart was then connected to a Langendorff apparatus and perfused by Tyrode’s solution with 95% O2 and 5% CO2 to maintain a pH of 7.35~7.45. A bipolar left atrial (LA) electrogram (LAE) and a pseudo-electrogram (p-ECG) were monitored. The p-ECG was obtained using widely spaced bipolar electrodes located in the bath surrounding the heart. The electrical signals were filtered from 0.05 to 100 Hz and digitized at 1 kHz using AxoScope (Molecular Devices, Downingtown, PA).

### Optical mapping

Optical mapping techniques^21^ were used to study the Vm of intact heart. The perfused Tyrode’s solution was maintained at 37.5 °C with a constant flow rate ~2 ml/min. After 20 minutes of stabilization, the hearts were stained with 20 μL of the voltage-sensitive dye RH237 (2.5 mmol/L) for Vm mapping. The heart was then washed for 10 min followed by the addition of blebbistatin (15 mmol/L) (Tocris Bioscience, Minneapolis, MN). The stained hearts were illuminated with a LED at a 520-nm wavelength and the fluorescence was collected by a MiCAM05-Ultima CMOS camera (BrainVision, Tokyo, Japan) through a 715-nm long-pass filter. The fluorescence signal was recorded at 1 ms/frame in a 100 × 100 pixel grid with a spatial resolution of 0.06 × 0.06 mm^2^ per pixel. Optical signals were processed with both spatial (3 × 3 pixels Gaussian filter) and temporal (3 frames moving average) filtering. The fluorescence obtained through a common lens was separated with a dichroic mirror (650 nm cutoff wavelength), and directed to the respective camera with additional filtering (715 nm long pass for Vm).

### HL-1 atrial myocyte culture

A murine HL-1 atrial myocyte cell line was maintained with fresh Claycomb medium in pre-coating culture flasks at 37 °C in a humidified atmosphere containing 5% CO2. When the cells reached confluence, splitting was performed by recommended passaging procedures. Culture medium was supplemented with 87% Claycomb medium, 2 mM/L L-glutamine, 10% fetal bovine serum, 100 U/ml penicillin, 100 µg/ml streptomycin, and 0.1 mM/L norepinephrine. To measure effects of VLDLs on expression and O-GlcNAcylation of Cx40 and Cx43, HL-1 cells were seeded overnight and then treated with Normal-VLDL or MetS-VLDL at a concentration of 25 mg/L for 16 h (n = 4 for each group).

### Detection of O-GlcNAcylated Cx40 and Cx43 proteins with immunoprecipitation

Protein extract was isolated from HL-1 cells using lysis buffer. Protein of 1 mg was mixed with anti-Cx40 (1:100 dilution, sc-20466, Santa Cruz) or anti-Cx43 (1:100 dilution, 15386-1-AP, Proteintech) at 4 °C for 1 h and then was incubated with Pierce™ Protein A/G Magnetic Beads (88802, Thermo Fisher Scientific) overnight at 4 °C. The IP matrix was washed twice by PBS containing 1% Nonidet P-40. The matrix-bound protein was eluted in sample buffer and then separation by 10% SDS-PAGE electrophoresis. The immunoblot was used the anti-OlcNAc (1:1000 dilution, MA1-076, Thermo Fisher Scientific), anti-Cx40 (1:500) or anti-Cx43 (1:1000) antibody overnight at 4 °C and signals was detected with secondary antibodies and chemiluminescence visualization.

### Structure prediction of the O-GlcNAcylated Cx40 and Cx43

The three-dimensional structures of human Cx40 and Cx43 were predicted by using the MODELLER 9v8^39^. For this study, the X-ray crystal structure of the Connexin-26 (Cx26) gap junction protein (PDB code 2ZW3) was selected as the template structure^40^. The models of Cx40 and Cx43 were built using this template. The O-GlcNAcylation sites of Cx40 and Cx43 were predicted by OGTSite^41^, which is a web server for identifying O-GlcNAcylation sites with their corresponding OGT substrate motifs. The structures of the O-GlcNAcylated Cx40 and Cx43 variants were built by using the GLYCAM online tool “Glycoprotein Builder”^42^. The “Glycoprotein Builder” was used to select the glycosylation site and attach the glycan to proteins to form either O- or N-linked glycoproteins. After obtaining the initial 3D model of a given glycosylated variant, an initial energy minimization of the glycoprotein was performed using a steepest descent algorithm^43^. Finally, the electrostatic potential surface of the glycoprotein was built using Glycan Reader^44^.

### Data analysis and statistics

The data was expressed as means ± SD unless indicated otherwise and n indicates the number of cell samples or mice. One-way ANOVA and Tukey’s multiple comparisons test were used to compare values among groups (Prism; GraphPad). Statistical significance was considered as p value ≤ 0.05.

## Electronic supplementary material

Supplementary material
