# Supplementary material for: Very-Low-Density Lipoprotein of Metabolic Syndrome Modulates Gap Junctions and Slows Cardiac Conduction
Source: Sci Rep. 2017 Sep 21;7:12050. doi: 10.1038/s41598-017-11416-5 (PMC5608762; doi:10.1038/s41598-017-11416-5)

Very-Low-Density Lipoprotein of Metabolic Syndrome Modulates Gap Junctions and Slows Cardiac Conduction

Hsiang-Chun Lee, MD, PhD; Chih-Chieh Chen, PhD; Wei-Chung Tsai, MD; Hsin-Ting Lin, MSc; Yi-Lin Shiao, MSc; Sheng-Hsiung Sheu, MD; Bin-Nan Wu, PhD; Chu-Huang Chen, MD, PhD; Wen-Ter Lai, MD

Supplementary material

The full-length gels and blots for figure 2.

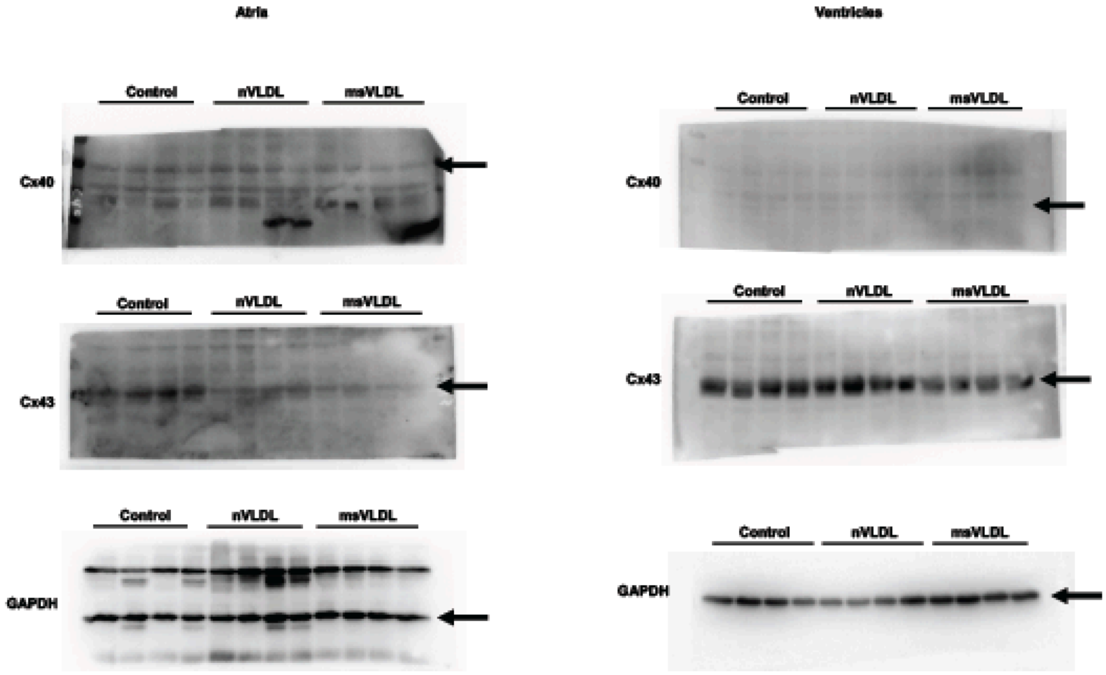

Supplement: Supplementary file 1 — Supplementary material [file 41598_2017_11416_MOESM1_ESM.pdf]
